# P-1017. Initial Results From a Real-World Patient Registry Study of Adults Receiving Fecal Microbiota, Live-jslm for the Prevention of Recurrent Clostridioides difficile Infection: The RebyOtA Prospective Registry (ROAR)

**DOI:** 10.1093/ofid/ofaf695.1213

**Published:** 2026-01-11

**Authors:** Nicholas W Van Hise, Teena Chopra, Kelly R Reveles, Sahil Khanna, Lasse Nielsen, Lorien E Urban

**Affiliations:** Metro Infectious Disease Consultants, Burr Ridge, IL; Detroit Medical Center, Wayne State University, Detroit, MI; The University of Texas at Austin, Austin, Texas; Mayo Clinic, Rochester, MN; Ferring Pharmaceuticals A/S, Kastrup, Hovedstaden, Denmark; Ferring Pharmaceuticals, Inc, Parsippany, New Jersey

## Abstract

**Background:**

Fecal microbiota, live-jslm (RBL) is FDA-approved for prevention of recurrent *Clostridioides difficile* infection (rCDI) in adults following completion of antibiotic treatment. The safety and efficacy of RBL have been demonstrated in trials, but few studies have evaluated outcomes in real-world populations. The primary objective of ROAR (NCT05835219) is to assess RBL effectiveness in reducing rCDI through 8 weeks in clinical practice.

**Table 1.**
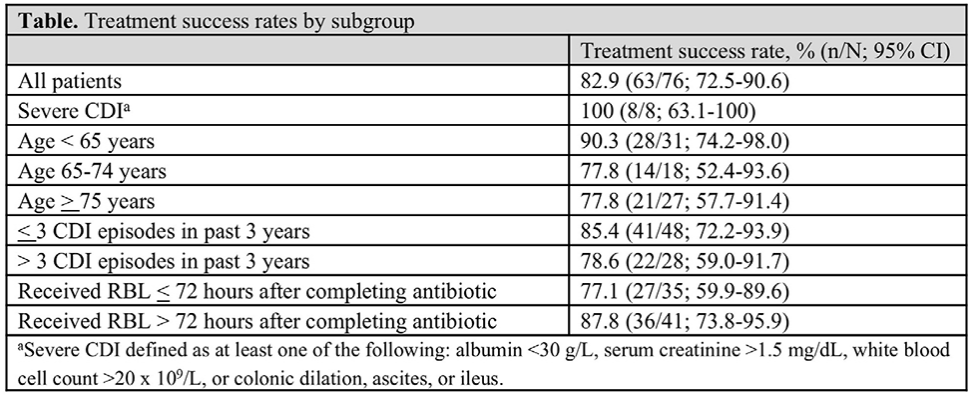
Treatment success rates by subgroup

**Methods:**

ROAR is an ongoing, prospective, multicenter, open-label, non-interventional registry of patients receiving RBL for rCDI prevention in the United States. Adults who completed antibiotics for rCDI and are not in an interventional trial are eligible. The primary endpoint assesses the proportion of patients who experience treatment success (no CDI recurrence through 8 weeks). Other objectives describe rCDI management in real-world clinical practice and adverse events (AEs) after RBL administration. This analysis includes patients who received RBL within 30 days of completing antibiotics for rCDI.

**Results:**

As of September 18, 2024, 76 patients received RBL within 30 days of completing antibiotics and completed 8 weeks of follow-up. Patients were mostly White (93.4%) and female (76.3%), with a median age of 69 years (range, 19-96 years) and a median body mass index of 24.4 (range, 16.5-49.4). Through 8 weeks, 82.9% of patients (63/76; 95% CI, 72.5%-90.6%) experienced treatment success. Similarly, the rate of treatment success was 87.8% (36/41) in the subgroup of patients who received RBL after an antibiotic washout period > 72 hours. Treatment success rates ranged from 77-100% across patient subgroups, including those stratified by age and number of previous CDI episodes within the past 3 years (Table). Overall, 18 (23.7%) patients experienced an AE, 6 (7.9%) experienced a serious AE, and 1 (1.3%) experienced an AE of special interest (large bowel obstruction). AEs assessed as related to RBL occurred in 3 patients (3.9%). There was 1 fatal AE during the study, considered unrelated to RBL.

**Conclusion:**

Initial results from ROAR are consistent with previous RBL trial results, indicating that RBL is effective and safe for preventing rCDI through 8 weeks after administration in real-world practice.

**Disclosures:**

Nicholas W. Van Hise, PharmD, Ferring: Advisor/Consultant|Ferring: Grant/Research Support|Innoviva: Advisor/Consultant Teena Chopra, MD, MPH, Cepheid: Advisor/Consultant|Ferring Pharmaceuticals, Inc: Advisor/Consultant|Melinta: Speaker|Pfizer: Advisor/Consultant|Pfizer: Speaker|Shinogi: Speaker Kelly R. Reveles, PharmD, PhD, AstraZeneca: Advisor/Consultant|Ferring Pharmaceuticals: Advisor/Consultant Sahil Khanna, MBBS, MS, Ferring Pharmaceuticals, Inc: Grant/Research Support|ProbioTech Inc: Advisor/Consultant|Rise: Advisor/Consultant|Takeda: Advisor/Consultant|Vedanta: Grant/Research Support Lasse Nielsen, MS, Ferring: Employee Lorien E. Urban, PhD, Ferring Pharmaceuticals Inc: Employee

